# RNA-binding protein LIN28B inhibits apoptosis through regulation of the AKT2/FOXO3A/BIM axis in ovarian cancer cells

**DOI:** 10.1038/s41392-018-0026-5

**Published:** 2018-08-31

**Authors:** Xiaojuan Lin, Jianfeng Shen, Dan Peng, Xinhong He, Congjian Xu, Xiaojun Chen, Janos L. Tanyi, Kathleen Montone, Yi Fan, Qihong Huang, Lin Zhang, Xiaomin Zhong

**Affiliations:** 1 Center for Research on Reproduction and Women’s Health, University of Pennsylvania, Philadelphia, PA 19104 USA; 2 Department of Gynecology and Obstetrics, Key Laboratory of Obstetrics & Gynecologic and Pediatric Diseases and Birth Defects of Ministry of Education, West China Second Hospital, Sichuan University, Chengdu, 610041 P.R. China; 3 Key Laboratory for Stem Cells and Tissue Engineering, Ministry of Education, Center for Stem Cell Biology and Tissue Engineering, Department of Biology, Zhongshan School of Medicine, Sun Yat-Sen University, Guangzhou, 510080 P.R. China; 4 Beijing Friendship Hospital, Capital Medical University, Beijing, 100050 P.R. China; 5 Obstetrics and Gynecology Hospital of Fudan University, Shanghai, 200011 P.R. China; 6 Department of Obstetrics and Gynecology, University of Pennsylvania, Philadelphia, PA 19104 USA; 7 Department of Pathology and Laboratory Medicine, University of Pennsylvania, Philadelphia, PA 19104 USA; 8 Department of Radiation Oncology, University of Pennsylvania, Philadelphia, PA 19104 USA; 9 Shanghai Respiratory Research Institute, Shanghai, 200032 P. R. China; 10 Department of Pulmonary and Critical Care Medicine, Zhongshan Hospital, Fudan University, Shanghai, 200032 P. R. China; 11 Institute of Clinical Science, Zhongshan Hospital, Fudan University, Shanghai, 200032 P. R. China

## Abstract

LIN28B is an evolutionarily conserved RNA-binding protein that regulates mRNA translation and miRNA *let-7* maturation in embryonic stem cells and developing tissues. Increasing evidence demonstrates that *LIN28B* is activated in cancer and serves as a critical oncogene. However, the underlying molecular mechanisms of LIN28B function in tumorigenesis are still largely unknown. Here we report that LIN28B was expressed in over half of the patients with epithelial ovarian cancer who were examined (*n* = 584). Functional experiments demonstrated that LIN28B inhibited ovarian cancer cell apoptosis. Furthermore, we showed that the proapoptotic factor BIM played an essential role in the antiapoptotic function of LIN28B. RNA-IP microarray analysis suggested that LIN28B binds to mRNAs that are associated with the DNA damage pathway, such as *AKT2*, in ovarian cancer cells. By binding to *AKT2* mRNA and enhancing its protein expression, LIN28B regulated FOXO3A protein phosphorylation and decreased the transcriptional level of *BIM*, which antagonized the antiapoptosis activity of LIN28B. Taken together, these results mechanistically linked LIN28B and the AKT2/FOXO3A/BIM axis to the apoptosis pathway. The findings may have important implications in the diagnosis and therapeutics of ovarian cancer.

## Cancer: ovarian cancer oncogene forces cells to stay alive

Researchers in China have uncovered the molecular mechanism behind the activity of a gene related to ovarian cancer. Xiaomin Zhong’s team at Sun Yat-Sen University knocked down the gene, *LIN28B*, in cancer cell lines and discovered that this increased their response to a chemical, which induces programmed cell death (PCD). By contrast, increasing LIN28B expression reduced PCD sensitivity, leading to the conclusion that LIN28B inhibits PCD in cancer cells. Examining gene expression in the knockdown lines revealed that LIN28B suppresses the activity of the PCD-related gene *BIM.* Further experiments showed that this happens through the modulation of genes upstream of *BIM*. These results demonstrate that LIN28B acts by regulating a genetic pathway, which regulates PCD. Altogether, this improved understanding of LIN28B may help with the diagnosis and therapy of ovarian cancer.

## Introduction

LIN28 is an evolutionarily conserved and developmentally regulated RNA-binding protein that was first characterized as a critical modulator of the developmental timing in *Caenorhabditis elegans* (*C. elegans*).^1–3^ It has been reported that the LIN28 protein interacts with mRNAs and regulates their translation^4–7^ and/or stability.^8^ LIN28 also binds to the precursors or primary transcripts of certain microRNAs (miRNAs), thereby blocking their maturation.^9–13^ The mammalian genome encodes *LIN28A* and *LIN28B*,^14,15^ two homologs of the *C. elegans lin-28* gene, which may modulate gene expression by different molecular mechanisms.^16^ In mammals, the *LIN28* family plays a critical role in multiple physiological processes, e.g., skeletal myogenesis,^6^ neurogliogenesis,^17^ lymphopoiesis,^18^ germ-cell development,^19^ and glucose metabolism.^20^ Genome-wide association studies have indicated an association of the *LIN28B* locus and height, as well as the timing of menarche, in humans. This association has been faithfully phenocopied in a transgenic mouse model.^21,22^ LIN28 may also function as a critical factor regulating the pluripotency of embryonic stem (ES) cells.^23,24^ Combined with other pluripotency factors, such as OCT4, NANOG, and SOX2, LIN28A can assist in reprogramming somatic cells to induce pluripotent stem cells.^25^

During the developmental process, the expression of *LIN28A* and *LIN28B* is strictly limited in ES cells and developing tissues. Their expression level is downregulated significantly when cellular differentiation proceeds. Increasing evidence indicates that the* LIN28* family may play a critical role in tumorigenesis. First, the expression of *LIN28A* and *LIN28B* is aberrantly reactivated in multiple types of human cancers but is undetectable in the corresponding normal tissues.^16,26,27^ Second, LIN28A and LIN28B specifically block the maturation of let-7, a well-characterized tumor suppressor miRNA targeting multiple oncogenes.^28,29^ Third,* LIN28A* and *LIN28B* function as oncogenes by promoting malignant transformation,^26,27,30–33^ inducing metastasis,^27,34–36^ regulating inflammation,^16,27,37^ and maintaining cancer stem cells.^27,38–40^ Importantly, clinical epidemiological studies have indicated that the *LIN28* family is associated with clinical outcomes in cancer patients,^41^ as well as with susceptibility to certain cancers.^42–44^

Epithelial ovarian cancer is the most frequent cause of gynecologic malignancy-related mortality in women, creating a pressing need to understand its genetic basis and identify molecular targets for therapy. Robust evidence from molecular epidemiological studies has suggested that LIN28B may play a critical role in this disease. First, both the LIN28B protein and mRNA are strongly expressed in ovarian cancer.^26,41,45^ Second, a high expression level of *LIN28B* is significantly associated with the risk of disease progression and death in ovarian cancer patients.^41^ Third, a polymorphism, rs12194974 (G > A) in the *LIN28B* promoter region, influences susceptibility to ovarian cancer.^42^ However, the underlying molecular mechanisms of *LIN28B* function in ovarian tumorigenesis are still largely unknown. In the present study, we mechanistically link LIN28B to the apoptosis pathway through regulation of the AKT2/FOXO3A/BIM axis in this disease.

## Results

### LIN28B protein expression in human epithelial ovarian cancer

**Fig. 1:**
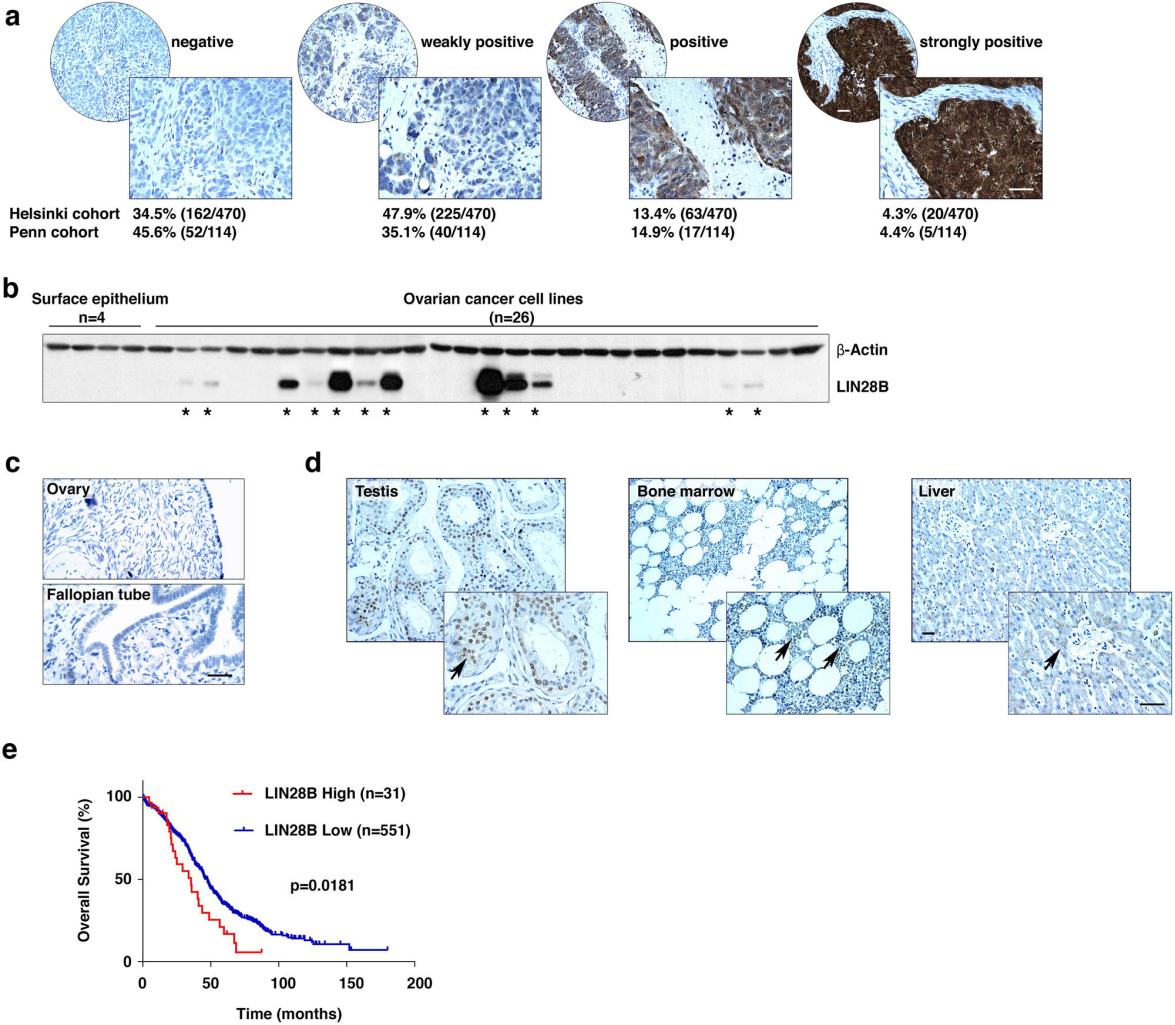
LIN28B expression and function in human epithelial ovarian cancer. **a** LIN28B expression in epithelial ovarian cancer specimens was detected by immunohistochemistry. Samples from two independent cohorts, the Helsinki cohort (*n* = 470) and the Penn cohort (*n* = 114), were used to statistically analyze the expression level of LIN28B protein (negative, weakly positive, positive, and strongly positive). Bar, 100 μm. **b** LIN28B expression in ovarian cancer cells was detected by western blotting. Four cultured human ovarian surface epithelial cell lines were used as controls. *LIN28B-positive cell lines. **c** Immunohistochemistry detected negative LIN28B expression in normal human ovarian surface and fallopian tube epithelia. Bar, 100 μm. **d** LIN28B expression was compared in testis (strongly positive), bone marrow, and liver (both weakly positive). Bar, 100 μm. **e** The overall survival curve of ovarian cancer patients grouped into *LIN28B* high and *LIN28B* low based on the mRNA expression level of *LIN28B*. Data were retrieved from the TCGA database. *n* = 582, *p* < 0.05

We examined the expression of LIN28B in two large collections of epithelial ovarian cancer specimens using immunohistochemistry. LIN28B was detected in more than 50% of these patient specimens (Helsinki cohort: 65.5%, 308/470; Penn cohort: 54.4%, 62/114, Fig. 1a). Strong LIN28B expression was found mainly in tumor cells (in both the cytoplasm and the nucleus), but not in stromal cells. This was confirmed by western blotting of 26 ovarian cancer cell lines (Fig. 1b). Importantly, LIN28B was undetectable in either the normal human ovarian surface or the fallopian tube epithelia, from which ovarian epithelial tumors may be derived (Fig. 1b, c). Furthermore, we also examined LIN28B expression in a Food and Drug Administration-approved normal human organ tissue microarray containing 24 types of organs. In adult tissues, strong LIN28B expression was found only in the testes, while weak expression was detected in the bone marrow and liver (Fig. 1d and Figure S1). The highly restricted expression of LIN28B in adult tissues suggests that LIN28B holds promise as a novel candidate for targeted therapy in developing new strategies for the treatment of ovarian cancer. Clinical data from The Cancer Genome Atlas database demonstrated a negative correlation between the* LIN28B* mRNA expression level and overall survival of 582 ovarian cancer patients (Fig. 1e), which further supported our hypothesis that *LIN28B* might act as an oncogene in the disease.

### LIN28B inhibits apoptosis in ovarian cancer cells

**Fig. 2:**
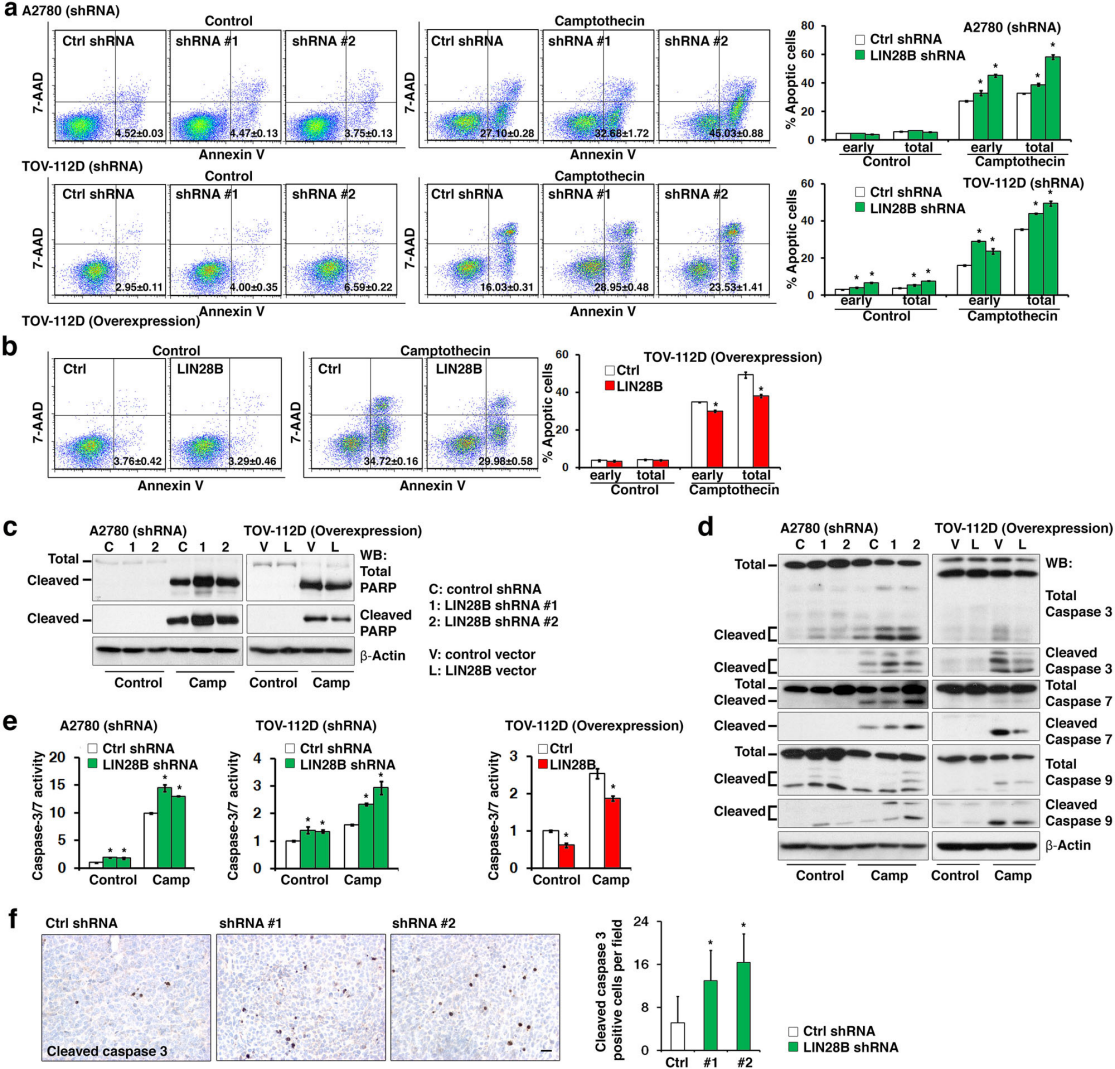
LIN28B inhibits apoptosis in ovarian cancer cells. **a** Inhibition of LIN28B expression by shRNAs significantly increased the number of apoptotic cells in A2780 (upper panel) and TOV-112D (lower panel) cells. An Annexin V assay was used to quantify the camptothecin-induced apoptotic cells. Statistical analysis of percentages of both early and total apoptotic cells is shown on the right. *p < 0.05. **b** Enforced expression of LIN28B significantly decreased the number of camptothecin-induced apoptotic cells in TOV-112D cells. An Annexin V assay was used to quantify the camptothecin-induced apoptotic cells. Statistical analysis of percentages of both early and total apoptotic cells is shown on the right. *p < 0.05. **c** Total and cleaved PARPs were detected by western blotting in A2780 (shRNA) and TOV-112D (overexpression) cells with or without camptothecin treatment. **d** Total and cleaved caspase-3, caspase-7, and caspase-9 were detected by western blotting in A2780 (LIN28B shRNA) and TOV-112D (LIN28B overexpression) cells with or without camptothecin treatment. **e** The caspase-3/7 enzyme activity was detected by a caspase-3/7 assay in A2780 (LIN28B shRNA), TOV-112D (LIN28B shRNA), and TOV-112D (LIN28B overexpression) cells with or without camptothecin treatment. *p < 0.05. **f** Knocking down LIN28B significantly increased the expression of cleaved caspase-3 detected by immunohistochemistry in A2780 xenograft tumors (left). Bar, 100 μm. The number of cleaved caspase-3-positive cells per field was analyzed and shown (right). *p < 0.05

To investigate the physiological functions of reactivated LIN28B in ovarian cancer, LIN28B was specifically knocked down in selected ovarian cancer cell lines (A2780: high LIN28B expression; TOV-112D: modest LIN28B expression; Figure S2a) using two independent lentiviral short hairpin RNAs (shRNAs). The shRNA-knockdown cell lines A2780 and TOV-112D were challenged with camptothecin, which is a common way to induce apoptosis in cancer cells, to evaluate the contribution of LIN28B to apoptosis. Camptothecin treatment led to significant increases in the percentage of apoptotic cells in both A2780 and TOV-112D cells with LIN28B shRNAs (Fig. 2a). In contrast, the enforced expression of LIN28B (Figure S2b) consistently reduced camptothecin-induced apoptosis in TOV-112D cells (Fig. 2b). These results were further confirmed by the detection of PARP cleavage using western blotting of A2780 (LIN28B shRNA) and TOV-112D (LIN28B overexpression) cells (Fig. 2c). We also examined total and cleaved caspase-3, -7, and -9 in A2780 (LIN28B shRNA) cells and found that blocking LIN28B expression led to increases in the cleaved forms of all three caspases (Fig. 2d). The phenotype was verified by enforced expression of LIN28B in TOV-112D cells, which showed decreased expression levels of the cleaved forms of caspase-3, -7, and -9 (Fig. 2d). To further confirm that not only the expression levels of the caspases *per se* but also the proteolysis activities of these caspases were affected by LIN28B, a caspase-3/7 enzymatic activity assay was performed. Consistent with the result in Fig. 2d, the activity of caspase-3/7 was increased in A2780 cells with LIN28B shRNAs, and it was decreased in TOV-112D cells with LIN28B overexpression (Fig. 2e). Finally, to functionally validate the in vivo effects of LIN28B on tumor cell apoptosis, we subcutaneously transplanted A2780 cells (LIN28B knockdown) and TOV-112D cells (LIN28B overexpression) into nude mice (Figure S3). The results showed that the size of the tumors formed was positively correlated with the expression level of LIN28B. LIN28B shRNA dramatically decreased the tumor size (Figure S3a, b), while LIN28B overexpression increased the tumor size (Figure S3c). To evaluate the contribution of apoptosis to the xenograft tumors formed, the percentage of apoptotic cells in A2780 (LIN28B knockdown) tumors, which was indicated by positive signals in immunohistochemistry staining for cleaved caspase-3, was detected and found significantly higher than that of the control tumors (Fig. 2f). These data suggested that LIN28B might promote tumor formation by suppression of apoptosis. Although LIN28B was shown to promote cell cycle progression by regulating G1/S transition (Figure S4a, b), the in vivo results of the xenograft tumor formation strongly supported the above in vitro data showing that LIN28B suppressed apoptosis in ovarian cancer cells. Taken together, all of the above evidence suggested a novel finding that *LIN28B* might function as an oncogene by suppressing apoptosis in ovarian cancer cells.

### LIN28B suppresses the expression of *BIM* to inhibit apoptosis

**Fig. 3:**
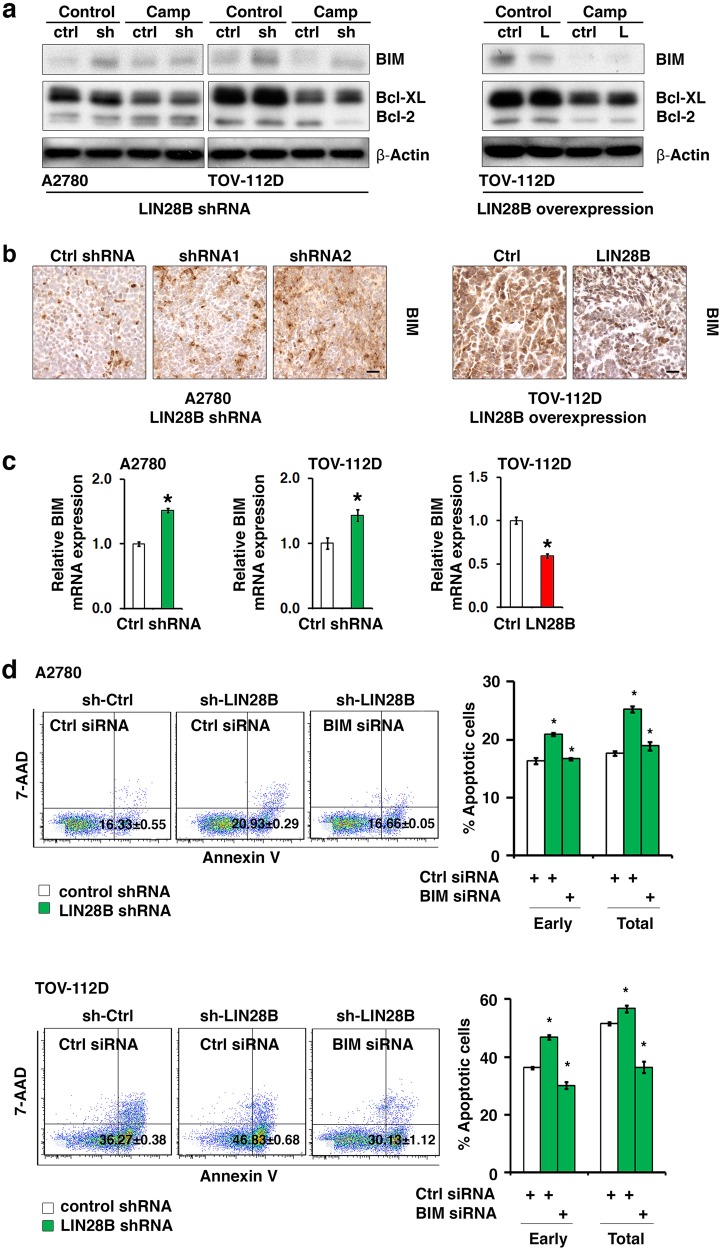
LIN28B suppresses the expression of *BIM* to inhibit apoptosis in ovarian cancer cells. **a** The expression of BIM, Bcl-XL, and Bcl-2 in LIN28B knockdown (A2780 and TOV-112D) and LIN28B enforced expression (TOV-112D) cells was examined by western blotting. Apoptosis was induced by camptothecin treatment. **b** The expression of BIM in xenograft tumors (A2780: LIN28B shRNA; and TOV-112D: LIN28B overexpression) was examined by immunohistochemistry. Bar, 100 μm. **c** The mRNA expression of *BIM* in LIN28B knockdown (A2780 and TOV-112D, left and middle) and LIN28B overexpression (TOV-112D, right) cells was examined by qRT-PCR. *p < 0.05. **d** Apoptosis induced by camptothecin was detected by an Annexin V assay in A2780 (top) and TOV-112D (bottom) cells with combined LIN28B and BIM knockdown. Representative data of three independent experiments with similar results are shown. Statistical analysis of percentages of both early and total apoptotic cells is shown on the right. *p < 0.05

To define the molecular mechanism through which LIN28B inhibited apoptosis, we examined the expression changes in selected key proteins in the apoptosis pathway, e.g., the proapoptotic factor BIM, and the antiapoptotic factors Bcl-XL and Bcl-2. We found that BIM protein expression was consistently activated upon LIN28B knockdown in both A2780 and TOV-112D cells, whether the cells were treated with camptothecin or not (Fig. 3a, left). Conversely, LIN28B overexpression in TOV-112D cells suppressed BIM expression (Fig. 3a, right). However, LIN28B knockdown and overexpression only had minor effects on the expression of Bcl-XL and Bcl-2 (Fig. 3a), indicating that BIM might be a critical mediator of the suppressive function of LIN28B on apoptosis. Furthermore, the regulation of LIN28B on BIM protein expression was validated by immunohistochemistry staining of xenograft tumor sections generated from A2780 (LIN28B shRNA) cells and TOV-112D (LIN28B overexpression) cells (Fig. 3b). To determine whether the regulation of BIM expression by LIN28B was at the transcriptional or translational level, the mRNA expression of *BIM* was detected upon LIN28B knockdown and overexpression. The transcript level of *BIM* showed expression changes similar to those of the BIM protein. LIN28B knockdown led to a significant increase in *BIM* mRNA level, while LIN28B overexpression resulted in a decrease in *BIM* mRNA level (Fig. 3c). However, the stabilities of both *BIM* mRNA and protein were unaffected by LIN28B (Figure S5a, b), suggesting the regulation of *BIM* expression by LIN28B was primarily at the transcriptional level. To further identify the functional role of BIM in apoptosis inhibition by LIN28B, BIM was knocked down by specific siRNA in A2780 and TOV-112D cells with LIN28B shRNA, and apoptosis was detected via Annexin V analysis (Fig. 3d). The results indicated that LIN28B shRNA alone increased the level of apoptosis as shown in Fig. 2. Interestingly, the addition of BIM siRNA to cells with LIN28B shRNA reversed the increase in the percentages of both early and total apoptotic cells caused by LIN28B knockdown to a level similar to that of the control cells with neither LIN28B shRNA nor BIM siRNA. These results indicated that BIM was a critical antagonist to apoptosis inhibition mediated by LIN28B. LIN28B suppressed apoptosis through downregulating the expression of *BIM* in ovarian cancer cells.

### LIN28B binds to mRNAs associated with the DNA damage pathway

**Fig. 4:**
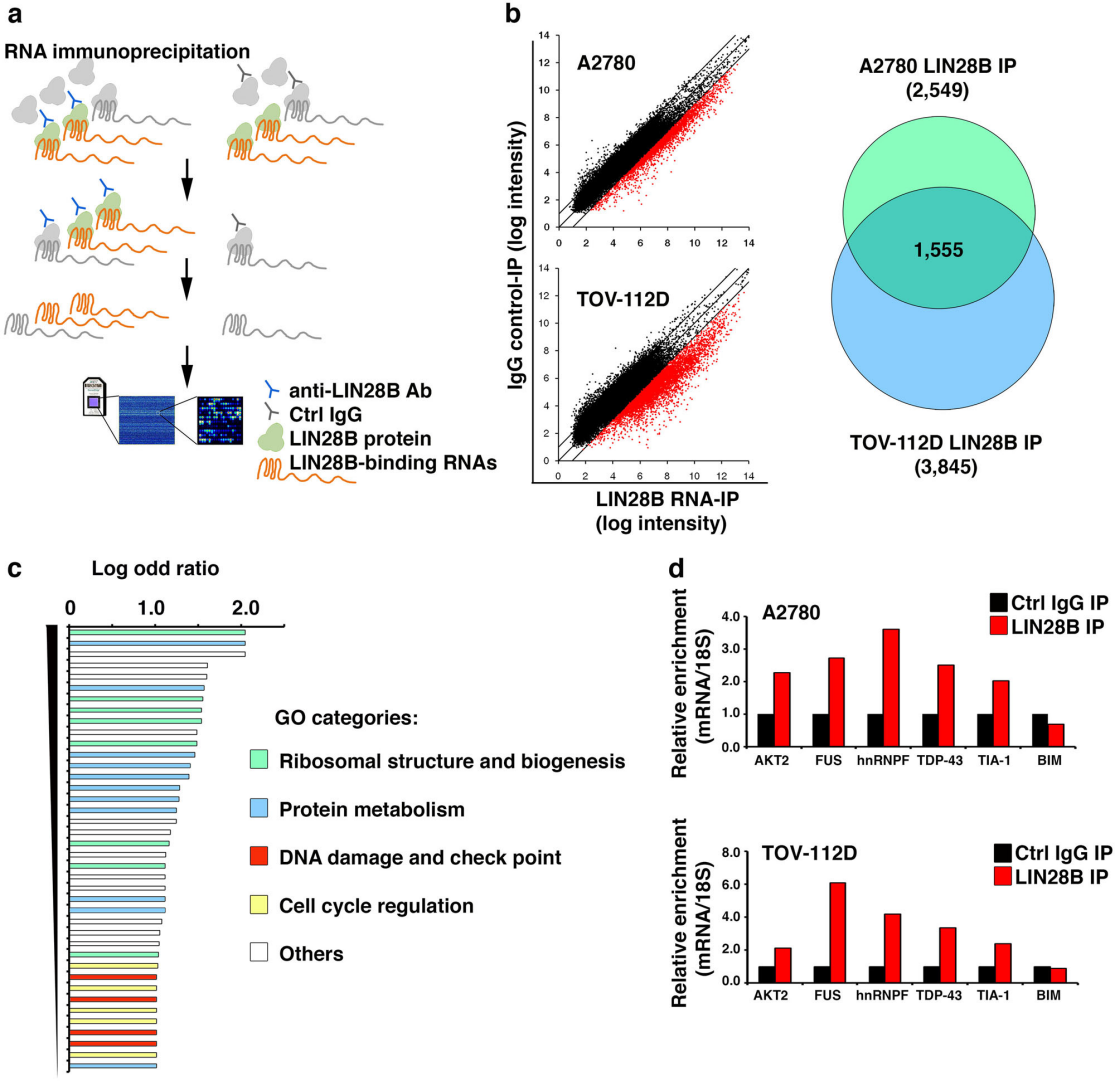
LIN28B binds to mRNAs associated with the DNA damage pathway. **a** RNA-IP microarray technology was used to identify genome-wide LIN28B-binding RNAs in ovarian cancer cells. **b** Dot plot (left) showed the enrichment fold of candidate RNA-binding partners of LIN28B in LIN28B RNA-IP compared to control IgG-IP samples. A total of 1555 mRNA transcripts enriched in the LIN28B RNA-IP sample were common in A2780 (total 2549) and TOV-112D (total 3845) cells (right). **c** GO analysis was performed on the transcripts bound by LIN28B. The major pathways identified by GO were ribosomal biogenesis, protein metabolism, DNA damage, and cell cycle pathways. **d** mRNA candidates from the DNA damage pathway enriched in LIN28B RNA-IP microarray were validated by independent RNA-IP experiments followed by qRT-PCR detection. The average data of two independent experiments with similar results are shown

As shown in the above results, LIN28B repressed *BIM* expression at the transcriptional level and directly affected the cellular sensitivity to apoptosis. However, the molecular mechanism utilized by LIN28B to regulate *BIM* expression remained unclear. It has been demonstrated that in most cases, LIN28B functions as an RNA-binding protein and contains two RNA-binding domains (a cold shock domain and two retroviral-type CCHC zinc finger motifs) that regulate the translation and/or stability of the target mRNAs.^5–7,46^ Based on this knowledge, it was hypothesized that LIN28B might bind to and regulate the mRNAs of some upstream factors controlling *BIM* transcription, rather than LIN28B directly repressing the transcriptional activity of the *BIM* gene by itself. To test this hypothesis, we performed RNA-immunoprecipitation (IP) microarray screening experiments (Fig. 4a) with two LIN28B-positive ovarian cancer cell lines (A2780 and TOV-112D). The RNP complexes containing the LIN28B protein and its associated RNAs were immunoprecipitated with a LIN28B-specific antibody. Subsequently, the Affymetrix gene expression microarray was used to profile the LIN28B-bound transcripts. We found that 1555 mRNAs were enriched in the LIN28B RNA-IP samples compared to the control IgG-IP samples from both A2780 and TOV-112D cells (Fig. 4b and Table S1). Subsequently, Gene Ontology (GO) analysis of these 1555 mRNAs was performed to explore the signaling pathways that might be controlled by LIN28B in ovarian cancer cells. As expected, the major pathways identified by the GO analysis included ribosomal biogenesis, protein metabolism, and cell cycle pathways (Fig. 4c and Table S2). This is consistent with a recent study of LIN28A RNA-IP deep sequencing in human ES cells,^46^ as well as previous reports demonstrating that LIN28A/B controlled G2/M transition in ES cells^23^ and promoted cancer cell proliferation.^47^ Interestingly, the DNA damage pathway associated with cancer cell apoptosis was also enriched in our RNA-IP experiments (Fig. 4c and Table S2), which might be a unique function of LIN28B. The mRNA candidates from the DNA damage pathway included *AKT2*, *FUS*, *hnRNPF*, *TDP-43*, and *TIA-1*. The interaction between the above mRNAs and the LIN28B protein could be validated repeatedly by independent experiments of RNA-IP followed by quantitative PCR detection (Fig. 4d), confirming the molecular association between LIN28B and the DNA damage pathway. In contrast,* BIM* mRNA was undetectable in the LIN28B-IP complex (Fig. 4d), suggesting that the modulation of *BIM* expression by LIN28B was probably an indirect effect.

### LIN28B regulates the protein expression level of AKT2

**Fig. 5:**
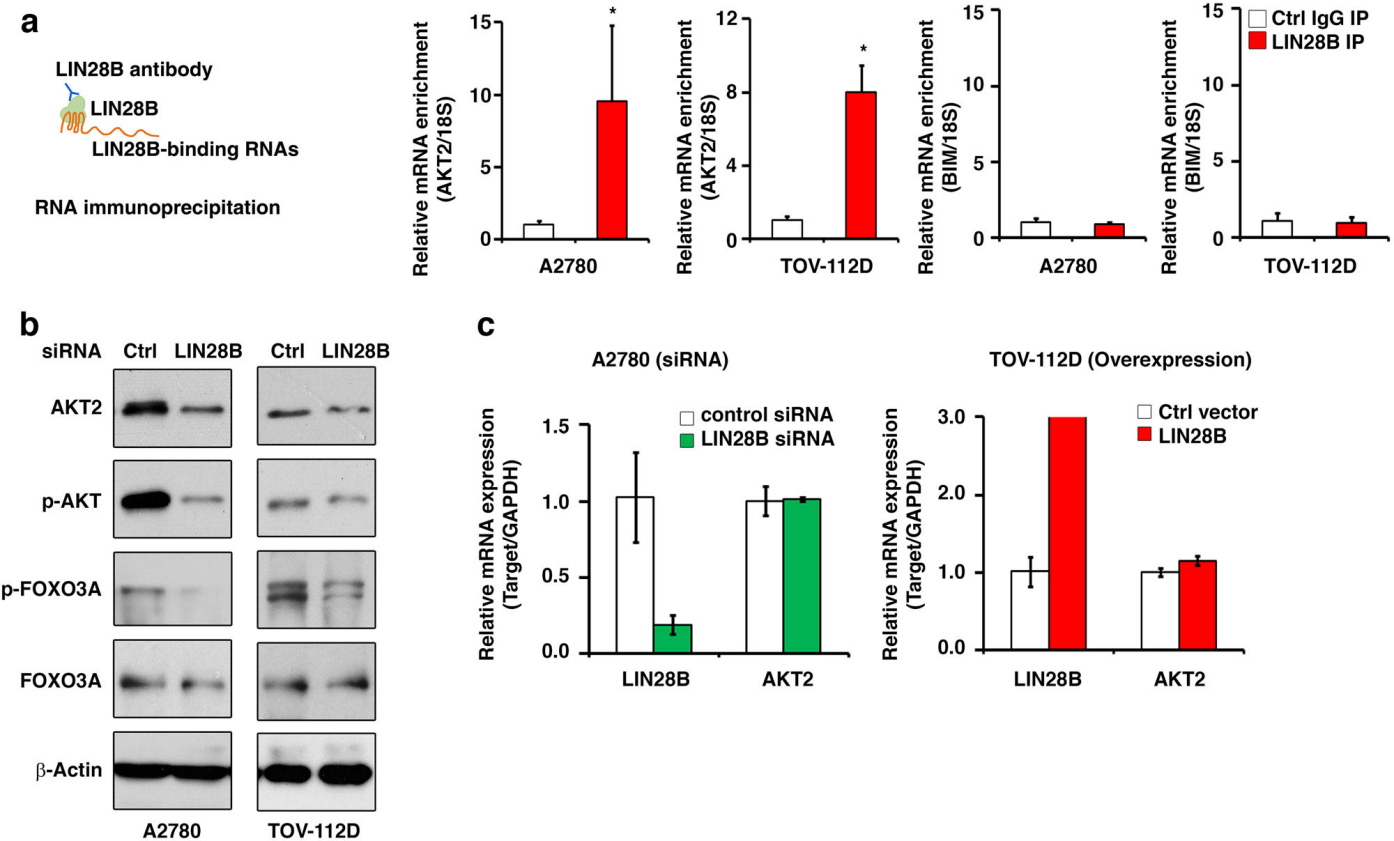
The protein expression level of AKT2 is regulated by LIN28B. **a** Detection of the binding of LIN28B to *AKT2* and *BIM* mRNA using RNA-IP followed by q-PCR assay in A2780 and TOV-112D cells. Bar graphs show representative results of relative mRNA enrichment of *AKT2* and *BIM* from three independent experiments. *p < 0.05. **b** The expression changes in total and phospho-AKT2 and total and phospho-FOXO3A upon LIN28B knockdown was detected by western blotting in A2780 and TOV-112D cells. **c** The mRNA level change in *AKT2*upon LIN28B knockdown (A2780) or overexpression (TOV-112D) was quantified by q-PCR. The results show the representative image from three independent experiments

Considering the above result indicating that the LIN28B protein directly associated with mRNAs of many key genes from the DNA damage pathway rather than *BIM* mRNA itself, we further hypothesized that the suppression of *BIM* expression by LIN28B might be a secondary effect of LIN28B binding and regulating the expression level of genes from the DNA damage pathway. To our interest, we found that one of the binding partners of LIN28B, *AKT2* (Fig. 4d), represented a well-known upstream regulator of *BIM* transcription. As widely reported, AKT2 can phosphorylate FOXO3A, the transcriptional activator of *BIM*, leading to FOXO3A translocation from the nucleus to the cytoplasm and inactivation of *BIM* transcription.^48^ Through this mechanism, the AKT2/FOXO3A/BIM axis has been demonstrated to be an important regulator of cellular apoptosis.^48,49^ Combined with our data, we suggested LIN28B might directly bind to *AKT2* mRNA and regulate *AKT2* expression to affect the transcription of *BIM*. To verify this hypothesis, we first confirmed the specific binding of LIN28B to *AKT2* mRNA, but not *BIM* mRNA, in both A2780 and TOV-112D cells by using a small-scale RNA-IP assay (Fig. 5a). Subsequently, the protein levels of AKT2 and FOXO3A were compared between the LIN28B knockdown cells and the control cells (Fig. 5b). As expected, both the total AKT2 and phospho-AKT2 protein levels were dramatically reduced upon LIN28B knockdown in A2780 and TOV-112D cell lines. Phospho-FOXO3A was also significantly diminished, which indicated a more stable form and a higher transcriptional activity of FOXO3A, by siRNA targeting of LIN28B. However, the mRNA level of *AKT2* was unaffected in either A2780 cells with LIN28B siRNA or TOV-112D cells with LIN28B overexpression (Fig. 5c). These results suggested that LIN28B might regulate the protein expression level of AKT2 by participating in *AKT2* mRNA transportation or the protein translation process. As a result, the interaction of LIN28B and AKT2 had a functional impact on the AKT2 pathway, as demonstrated by modulating the activity of AKT2 downstream factor FOXO3A, which might be responsible for the suppression of *BIM* expression.

### The AKT2/FOXO3A axis mediates the regulatory function of LIN28B on *BIM* expression

**Fig. 6:**
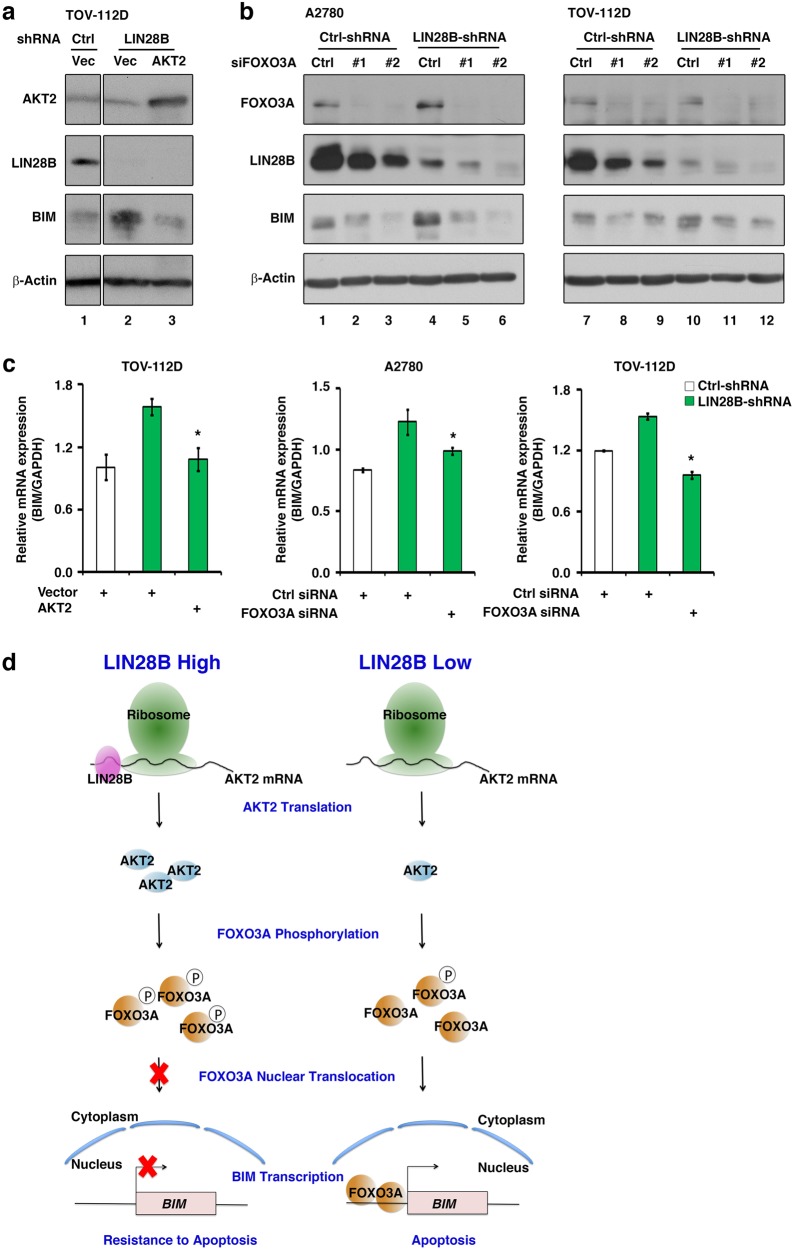
The AKT2/FOXO3A axis mediates the regulatory function of LIN28B on *BIM* expression. BIM protein level was detected by western blotting in TOV-112D cells with combined LIN28B knockdown and AKT2 overexpression. **b** BIM protein level was examined by western blotting in A2780 and TOV-112D cells with combined LIN28B knockdown and FOXO3A knockdown. **c** *BIM* mRNA level was detected by q-PCR upon AKT2 overexpression in TOV-112D (LIN28B shRNA) and upon FOXO3A knockdown in A2780 (LIN28B shRNA) and TOV-112D (LIN28B shRNA) cells. Representative results from three independent experiments were shown. *p < 0.05. **d** The mechanism governing the regulation of apoptosis by LIN28B through the AKT2/FOXO3A/BIM axis. Left: in cancer cells with a high expression level of LIN28B, the LIN28B protein associates with *AKT2* mRNA and enhances its translation efficiency. AKT2 phosphorylates FOXO3A and blocks the nuclear translocation of FOXO3A, which results in transcriptional silencing of the *BIM* gene and resistance to cellular apoptosis. Right: in cancer cells with a low expression level of LIN28B, the translation of *AKT2*mRNA is decreased. Phosphorylation of FOXO3A by AKT2 is also diminished. The nuclear translocation of FOXO3A leads to transcriptional activation of the *BIM* gene and cellular apoptosis

With an attempt to dissect the role of the AKT2/FOXO3A axis in the LIN28B regulation of *BIM* expression, we performed AKT2 overexpression or FOXO3A knockdown experiments to detect whether *BIM*expression can be rescued in LIN28B knockdown cells. In TOV-112D cells, LIN28B knockdown dramatically increased BIM protein levels (Fig. 6a, lane 2 compared to lane 1), whereas this effect could be reversed by the ectopic overexpression of an AKT2 plasmid (Fig. 6a, lane 3 compared to lane 2). Subsequently, we knocked down the expression of FOXO3A using two independent siRNAs in A2780 and TOV-112D cells. Notably, downregulation of FOXO3A mimicked the effect of AKT2 overexpression and reversed BIM protein expression to a lower level in both A2780 and TOV-112D cells with LIN28B shRNA (Fig. 6b, lanes 5 and 6 compared to lane 4, as well as lanes 11 and 12 compared to lane 10). Furthermore, we analyzed the mRNA expression of *BIM* under AKT2 overexpression or FOXO3A knockdown. Similar to the changes in BIM protein in Fig. 6a, b, the upregulation of *BIM* mRNA by LIN28B knockdown could be rescued by either AKT2 overexpression (Fig. 6c, left) or FOXO3A knockdown (Fig. 6c, middle and right), which confirmed the above result showing that LIN28B regulates *BIM* expression primarily at the transcriptional level. In summary, the modulation at both the mRNA and protein levels of *BIM*by the AKT2/FOXO3A axis under the cellular context of LIN28B knockdown demonstrated an essential role of AKT2/FOXO3A in mediating the regulatory function of LIN28B on *BIM* expression (Fig. 6d).

## Discussion

In the present study, we linked oncogenic protein LIN28B to the apoptosis pathway and identified the molecular mechanism for the antiapoptotic function of LIN28B in ovarian cancer cells. LIN28B suppressed *BIM* expression at the transcriptional level and conferred apoptotic resistance in ovarian cancer cells. The underlying mechanism for the regulation of *BIM* expression by LIN28B was further characterized. We identified the *AKT2* mRNA molecule as a binding partner of the LIN28B protein, and LIN28B enhanced the protein expression level of AKT2. Interestingly, AKT2 and its downstream target FOXO3A have been widely reported to form a regulatory axis for *BIM* gene transcription. Consistent with the previous knowledge, we found that the AKT2/FOXO3A axis mediated the inhibitory function of LIN28B on *BIM* expression and modulated cellular apoptosis.

This is the first report to experimentally associate LIN28B with the apoptosis pathway in ovarian cancer cells, as well as to discover the suppressive function of LIN28B on *BIM* expression. Specifically, the antiapoptotic function was unique to LIN28B rather than its ortholog LIN28A (data not shown). Our further mechanistic study revealed an essential role of the AKT2/FOXO3A axis in LIN28B-mediated apoptosis suppression via the modulation of *BIM* expression. The direct binding of *AKT2* mRNA and the LIN28B protein indicated that LIN28B might actively affect the translational efficiency of *AKT2* mRNA, which was supported by the change in protein level, instead of mRNA level, of *AKT2* upon LIN28B knockdown (Fig. 5). However, previous studies by other groups also reported that LIN28B can increase the activity of the PI3-kinase/AKT pathway by a *let-7*-dependent mechanism.^20^ Thus, LIN28B may activate the PI3-kinase/AKT pathway by blocking *let-7* and subsequently decrease *BIM* transcription. Since there is a possibility that *let-7* may be involved in the regulation of AKT expression by LIN28B, we hypothesize that it may be a synergistic, rather than controversial, mechanism that LIN28B not only represses *let-7* biogenesis to release the suppressive effect on AKT expression by *let-7* but also directly interacts with and enhances the protein translational efficiency of *AKT2* mRNA by itself. In addition, we would not exclude other mechanisms that may also be involved in the antiapoptotic function of LIN28B, since there was a large group of apoptosis-associated mRNAs binding to LIN28B (Fig. 4). Although many possibilities need to be explored to fully characterize the regulation of apoptosis by LIN28B, these possibilities all support that LIN28B functions as an antiapoptotic oncogene.

*LIN28B* had been identified to act as an oncogene by promoting malignant transformation,^26,27,30–33,50^ inducing metastasis,^27,34–36^ regulating inflammation,^16,27,37^ and maintaining cancer stem cells.^27,38–40^ Continuous efforts had been exerted to dissect the regulatory networks governed by LIN28B in human cancers. It was well demonstrated that LIN28B selectively represses certain miRNAs, including *let-7*,^10,11,50^ one of the first-identified tumor suppressor miRNAs,^28^ which controls the expression of hundreds of target genes. LIN28B may also bind directly to thousands of other mRNAs involved in various physiological processes and regulate their expression.^5–7,46^ Based on these facts, we speculate that the potential regulatory circuitry afforded by LIN28B in cancer cells is enormous and may be complex. For example, in addition to the apoptosis pathway, our RNA-IP microarray experiment also found that LIN28B directly binds to a large number of cell cycle-associated genes (Fig. 4c). We had experimentally validated this with an in vitro BrdU labeling analysis and found that LIN28B could promote cell cycle transition after serum starvation in ovarian cancer cells (Figure S4). Therefore, the oncogenic function of LIN28B may be context-dependent and cancer-type-specific, that is, the outcome of LIN28B reactivation in cancers largely depends on the existing cellular mRNA and miRNA transcriptome in different cancer patients. Functional characterization of the downstream molecular networks regulated by LIN28B will contribute to developing novel targeted therapeutic strategies for human cancer.

## Materials and methods

### Cell lines and cell culture

Ovarian cancer cell lines were obtained from the American Type Culture Collection and the Division of Cancer Treatment and Diagnosis Tumor/Cell Line Repository in 2009. All cancer cell lines were maintained in 10% fetal bovine serum (FBS, Invitrogen, CA)-supplemented RPMI 1640 medium (Invitrogen). Four immortalized human ovarian surface epithelial (IOSE) cell lines were generous gifts from Dr. Nelly Auersperg in 2009. IOSE cells were cultured in Media 199:MCDB 105 (Sigma) (1:1) supplemented with 15% FBS. Cell lines used in all the experiments were cultured no more than two passages between collection or thawing and use. (No cell authentication and mycoplasma testing were done.)

### Plasmids

The two independent shRNA sequences cloned into the lentiviral vectors targeting human *LIN28B* (pLKO.1, TRCN0000122191 and TRCN0000122599) were obtained from Open Biosystems. shRNA for enhanced green fluorescent protein (SHC005) and the nontarget shRNA (SHC002) served as negative controls. For studies using the constructed pMSCV-neo-*hLIN28B* retroviral expression vector (Addgene), the pMSCV-neo empty vector was used as a control.

### Antibodies

Primary antibodies for caspase-3, cleaved caspase-3, caspase-7, cleaved caspase-7, caspase-9, cleaved caspase-9, PARP, cleaved PARP, LIN28B, BIM, Bcl-XL, Bcl-2, AKT2, and phospho-AKT (Ser473) were all purchased from Cell Signaling.

### Tissue microarrays

The ovarian cancer tissue microarrays were constructed by the Helsinki University Central Hospital (Helsinki cohort) and the Hospital of the University of Pennsylvania (Penn cohort). The normal human tissue microarray was purchased from the Tissue Array Network.

### RNA-immunoprecipitation

The cells (5 × 10^6^) were lysed for 15 min on ice in a Polysome Lysis Buffer consisting of 100 mM KCl, 5 mM MgCl_2_, 10 mM HEPES (pH 7.0), and 0.5% NP-40 detergent freshly supplemented with 1 mM dithiothreitol (DTT), 100 U/ml RNase-Out (Invitrogen), 400 µM vanadyl-ribonucleoside complex (VRC) (New England Biolabs), and a protease inhibitor cocktail (Sigma). The cell lysate was diluted in 1:10 with NT2 Buffer consisting of 50 mM Tris-HCl (pH 7.4), 150 mM NaCl, 1 mM MgCl_2_, and 0.05% NP-40 freshly supplemented with 200 U/ml RNase-Out, 400 µM VRC, 1 mM DTT, 20 mM EDTA, and a protease inhibitor cocktail. The insoluble particles in the lysate were precipitated by centrifugation at 15 000 × *g* for 15 min at 4 °C. The LIN28B antibody (1:75) or control IgG was added to protein-A Sepharose beads (Sigma) preincubated in 5% bovine serum albumin-NT2 Buffer for 1 h at 4 °C. After gentle rotation for 4 h at 4 °C, the beads were washed four times in cold NT2 Buffer and added to the cell lysates (10 µl beads/ml lysate). IP was performed by gentle rotation overnight at 4 °C. The immunoprecipitated complexes were washed four times in NT2 Buffer and resuspended in 100 µl NT2 Buffer containing 30 µg proteinase K (QIAGEN) to release the RNP complex. RNAs from the immunoprecipitates were extracted with the TRIzol reagent.

### Transfection of siRNA

siRNA, shRNA, and control oligonucleotides were purchased from IDT. The sequences were listed as following: siLIN28B (gggagauagaugcuacaacuguggt); siBIM#1 (ucccuacagacagagccacaagaca); siBIM#2 (accaccacuugauucuugcagccac); and siRNA control (nontargeting control). Transfections were done using the Lipofectamine™ RNAiMAX transfection reagent (Invitrogen) according to the manufacturer’s guides. Cells were then incubated in media containing the transfection mixture for 72 h before harvesting.

### Annexin V apoptosis assays

Annexin V staining using an apoptosis detection kit (R&D Systems) was detected through flow cytometry according to the manufacturer’s instructions.

### Caspase-3/7 activity assay

Caspase-3/7 activity assays were performed in 96-well plates using an Apo-ONE Homogeneous Caspase-3/7 Assay kit (Promega) following the manufacturer’s instructions. The resulting fluorescent intensity was quantified using a Fluoroskan Ascent FL microplate reader (Thermo Scientific).

### Generation of in vivo xenograft model

Female nude mice of 6–8 weeks of age were purchased from The Jackson Laboratory. Ovarian cancer cells (2.5 × 10^6^) were suspended in a total volume of 0.1 ml phosphate-buffered saline and were injected subcutaneously into the mouse flank. Tumors were detectable after approximately 10 days, and the tumor size was measured using a Vernier caliper. Tumor volumes were calculated using the formula *V* = ½ (*L* × *W*)^2^, where *L* is the length (longest dimension) and *W* is the width (shortest dimension) of the tumor. The animal study protocol was reviewed and approved by the Institutional Animal Care and Use Committee of the University of Pennsylvania.

### Detecting the stabilities of RNA and protein

The decay rate of BIM mRNA was measured using a quantitative real-time PCR (qRT-PCR)-based time-course analysis. Briefly, actinomycin D (10 µg/ml, Sigma) was added to the culture medium to block transcription. The cells were then harvested, and total RNA was extracted at 0, 3, 6, 9, and 12 h post actinomycin D treatment. qRT-PCR was used to determine the levels of BIM mRNA. The mRNA decay curve was plotted by setting 0 h as 100%. To measure the half-life of endogenous BIM protein, cells were treated with 100 μg/ml cycloheximide (Sigma) and harvested at 0, 2, 4, 8, and 12 h post cycloheximide treatment. Proteins were detected by western blotting.

### Bioinformatics analysis

FuncAssociate was applied to search for GO attributes.

### Statistical analysis

Statistical analysis was performed using SPSS and SAS statistical software packages. All results were expressed as the mean ± SD, with *p* < 0.05 indicating statistical significance.

## Electronic supplementary material

Supplemental Table S2

Supplemental Figures and Legends

Supplemental Table S1
