# Supplementary material for: RNA-binding protein LIN28B inhibits apoptosis through regulation of the AKT2/FOXO3A/BIM axis in ovarian cancer cells
Source: Signal Transduct Target Ther. 2018 Aug 31;3:23. doi: 10.1038/s41392-018-0026-5 (PMC6117292; doi:10.1038/s41392-018-0026-5)
Supplement: Supplementary file 2 — Supplemental Figures and Legends [file 41392_2018_26_MOESM2_ESM.docx]

**Supplemental Figures**

**RNA-binding protein LIN28B inhibits apoptosis through regulating the AKT2/FOXO3A/BIM axis in ovarian cancer cells**

Xiaojuan Lin^1,2†^, Jianfeng Shen^1†^, Dan Peng^3^, Xinhong He^1,4^, Congjian Xu^5^, Xiaojun Chen^5^, Janos L Tanyi^6^, Kathleen Montone^7^, Yi Fan^8^, Qihong Huang^9^, Lin Zhang^1, 6^, Xiaomin Zhong^3*^

1. Center for Research on Reproduction and Women's Health, University of Pennsylvania, Philadelphia, PA 19104;

2. Department of Gynecology and Obstetrics, Key Laboratory of Obstetrics & Gynecologic and Pediatric Diseases and Birth Defects of Ministry of Education, West China Second Hospital, Sichuan University, Chengdu, China, 610041;

3. Key Laboratory for Stem Cells and Tissue Engineering, Ministry of Education, Center for Stem Cell Biology and Tissue Engineering, Department of Biology, Zhongshan School of Medicine, Sun Yat-Sen University, Guangzhou, China 510080;

4. Beijing Friendship Hospital, Capital Medical University, Beijing, China 100050;

5. Obstetrics and Gynecology Hospital of Fudan University, Shanghai, China 200011;

6. Department of Obstetrics and Gynecology, University of Pennsylvania, Philadelphia, PA 19104;

7. Department of Pathology and Laboratory Medicine, University of Pennsylvania, Philadelphia, PA 19104;

8. Department of Radiation Oncology, University of Pennsylvania, Philadelphia, PA 19104;

9. Wistar Institute, Philadelphia, PA 19104.

^†^These authors contributed equally to this work.

*Corresponding author: Xiaomin Zhong, zhongxm23@mail.sysu.edu.cn

**Figure S1**


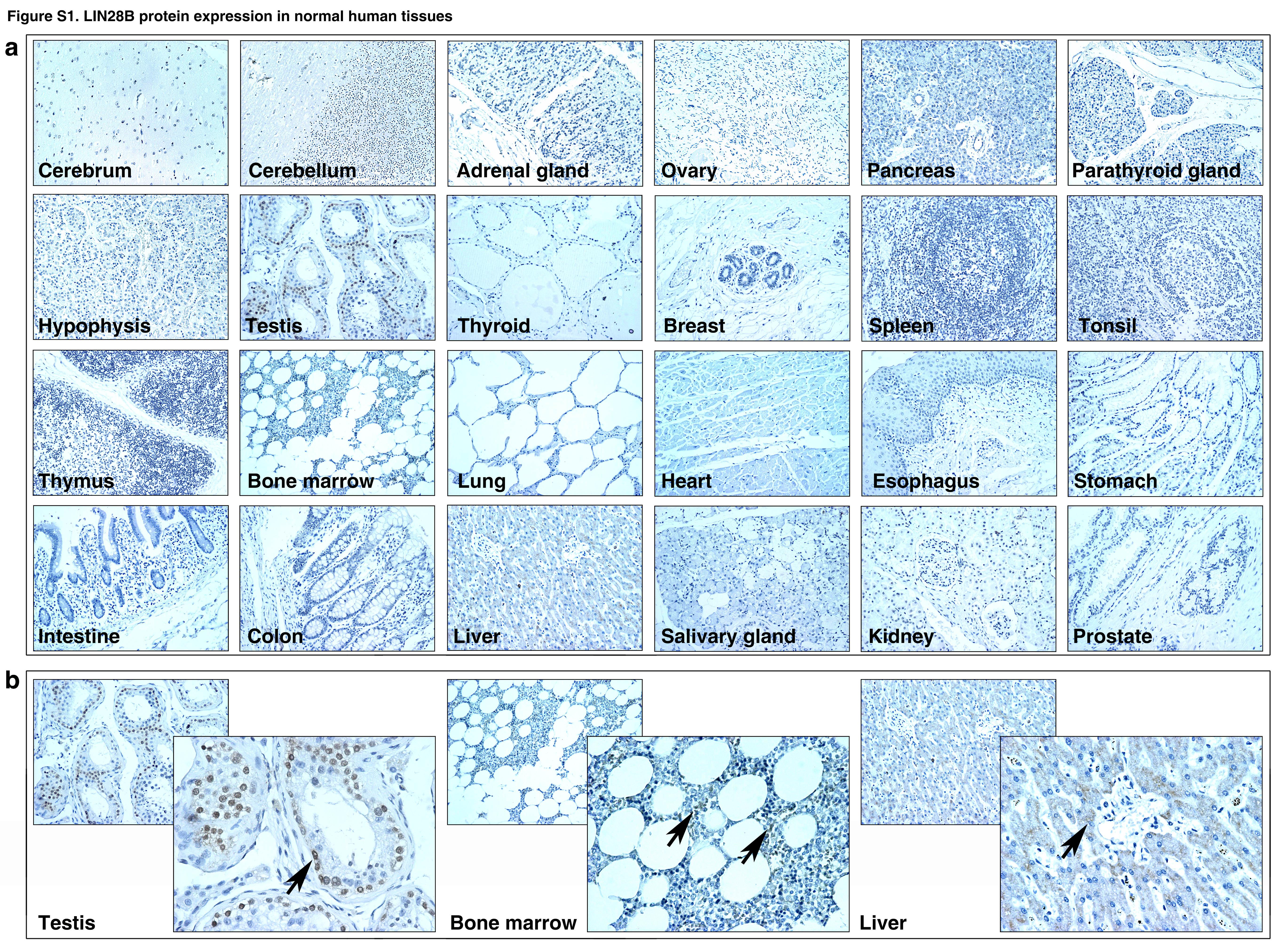


**Figure S1. LIN28B protein expression in normal human tissues**

**(a)** An **FDA approved** normal human organ tissue microarray was used to examine the expression of LIN28B in adult human tissues. There were 24 types of organs included in triplicate **on this array**, each from three separate individuals. **(b)** In adult tissues, strong LIN28B expression was seen in the testis, and weak expression was detected in the bone marrow and liver.

**Figure S2**


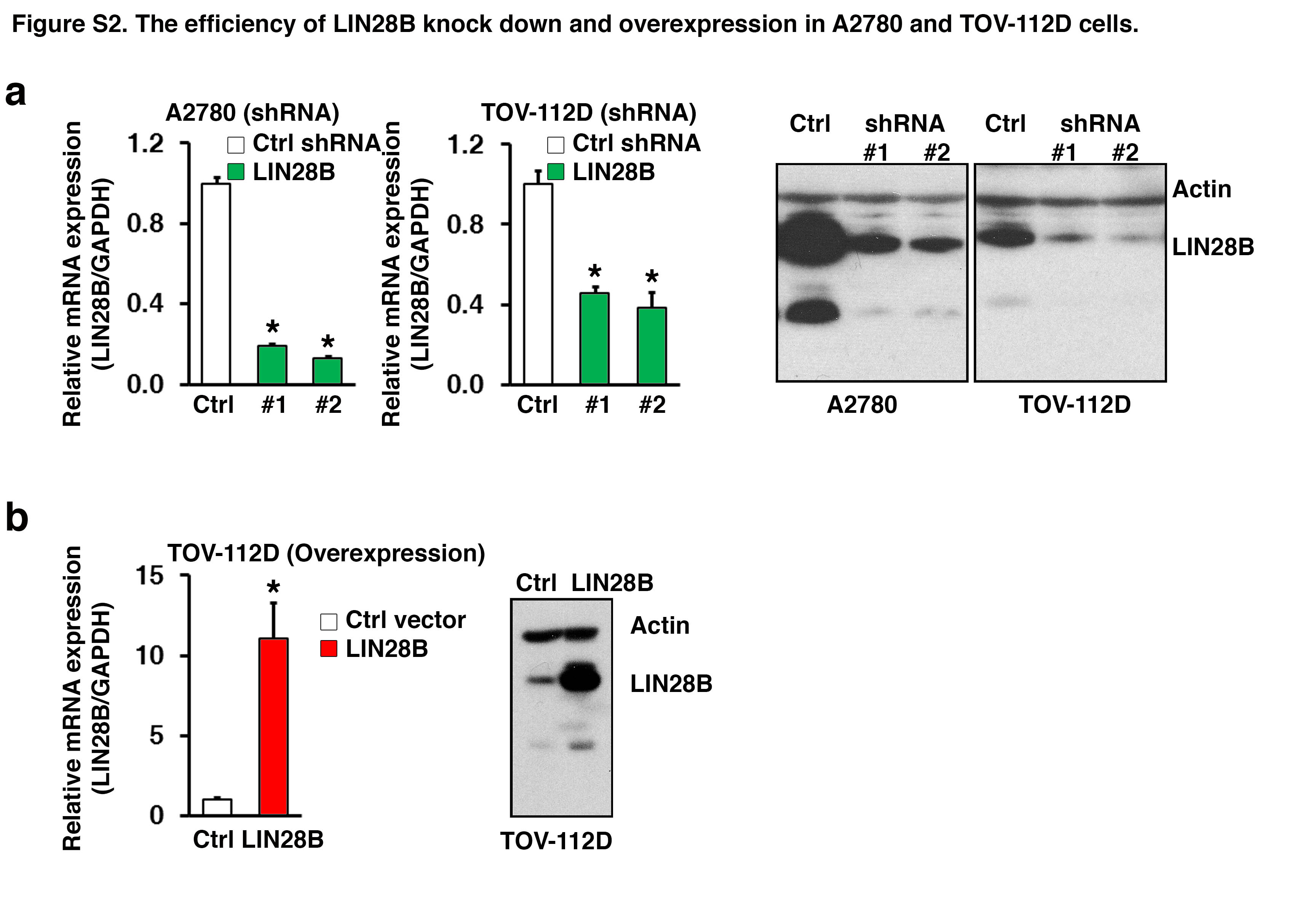


**Figure S2. The efficiency of LIN28B knock down and overexpression in A2780 and TOV-112D cells**

**(a)** LIN28B was specifically knocked down in ovarian cancer cell lines A2780 and TOV-112D using two independent lentiviral shRNAs. qRT-PCR (left) and Western blotting (right) were used to detect the knockdown efficiency of LIN28B. *, p < 0.05. **(b)** LIN28B expression was examined with qRT-PCR (left) and Western blotting (right) in TOV-112D cells overexpressing LIN28B. *, p < 0.05.

**Figure S3**


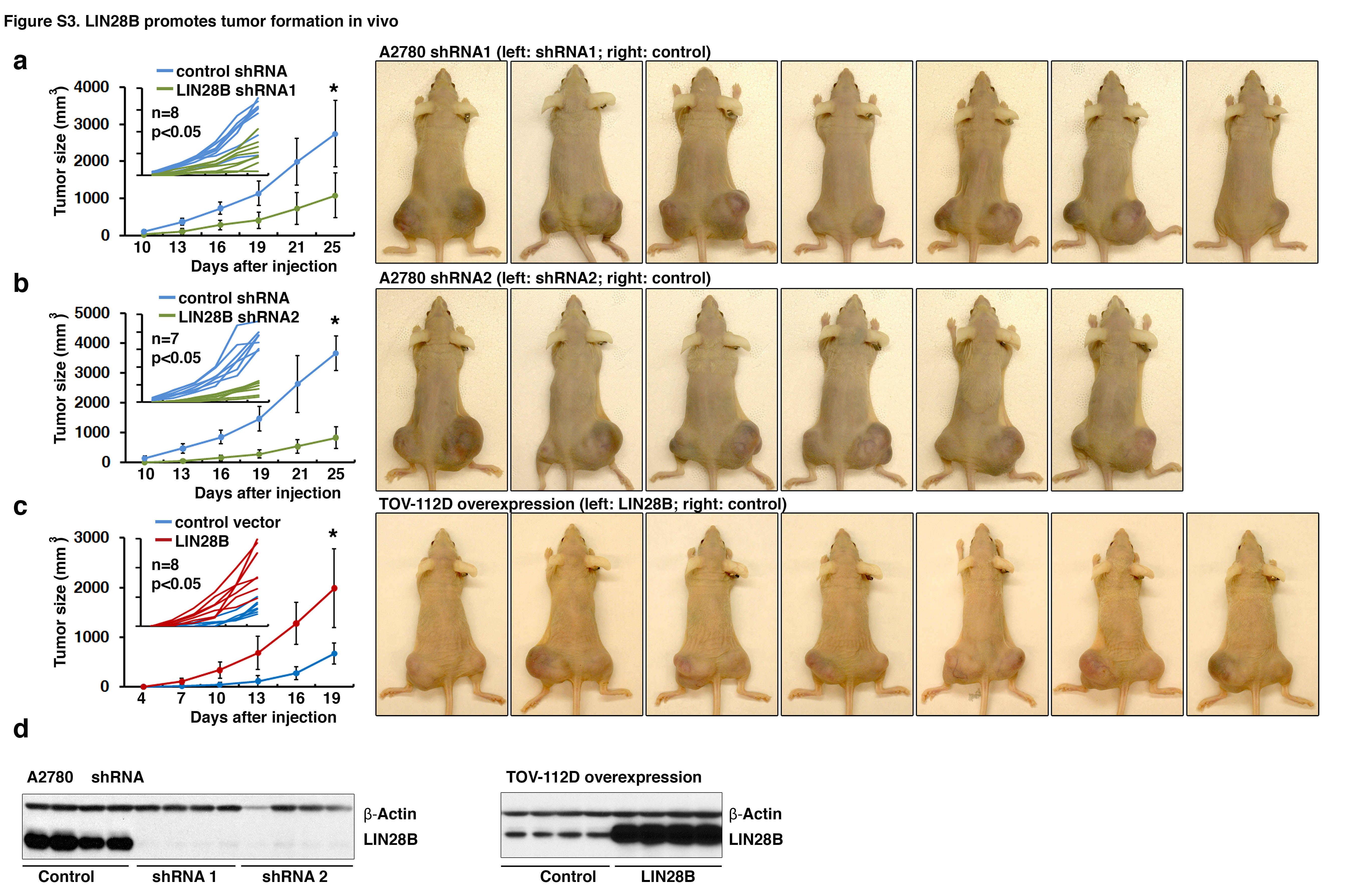


**Figure S3. LIN28B promotes tumor formation *in vivo***

**(a)** **and (b)** A2780 cells (LIN28B knockdown) were transplanted into nude mice. Statistical analysis of tumor size from LIN28B shRNA cells compared to control shRNA cells was shown in left. *, p < 0.05. **(c)** TOV-112D cells (LIN28B overexpression) were transplanted into nude mice. Statistical analysis of tumor size from LIN28B overexpressing cells compared to control vector cells was shown in left. *, p < 0.05. **(d)** Efficiency of LIN28B knockdown or overexpression in xenografted tumors was confirmed using Western blotting. *, p < 0.05.

**Figure S4**


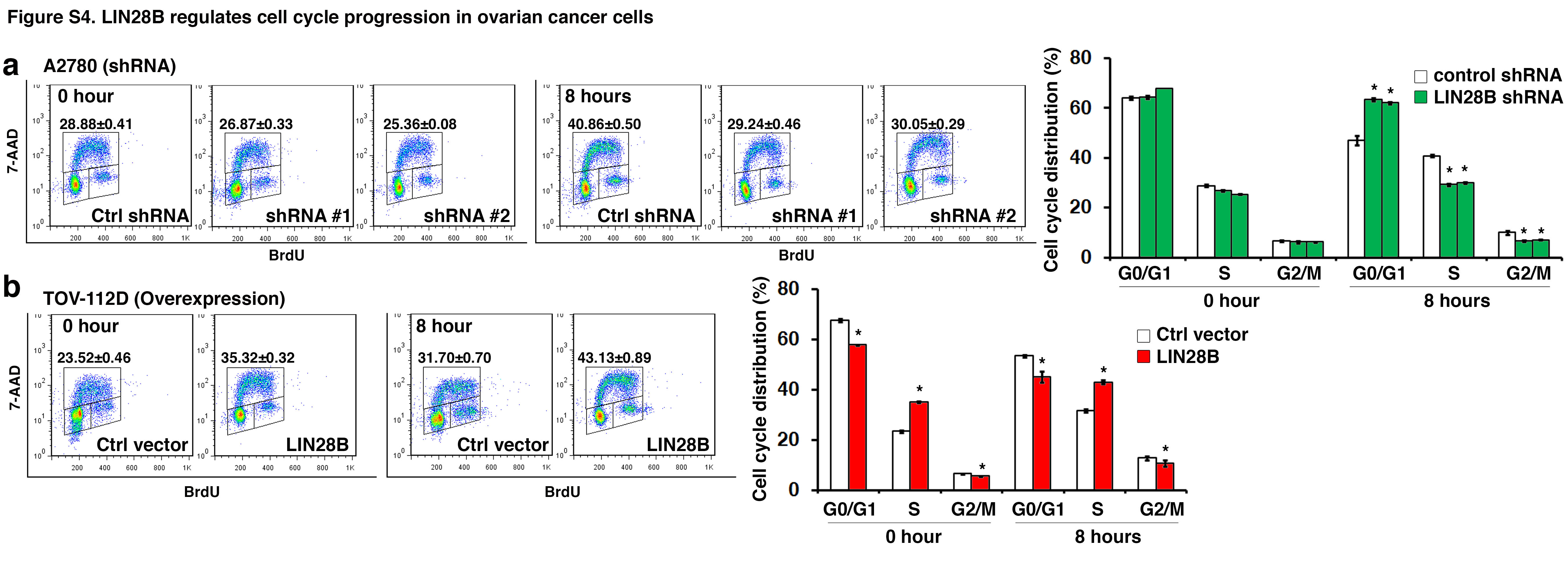


**Figure S4. LIN28B regulates cell cycle progression in ovarian cancer cells**

**(a)** Cell cycle distribution of A2780 cells with control shRNA or LIN28B shRNA was detected through BrdU labeling at 0 and 8hrs post-BrdU treatment. Left: representative data of flow cytometry from three independent experiments with similar results were shown. Right: statistical analysis of cell cycle distribution was shown. *, p < 0.05. **(b)** Cell cycle distribution of TOV-112D cells with control vector or LIN28B overexpression vector was detected through BrdU labeling at 0 and 8hrs post-BrdU treatment. Left: representative data of flow cytometry from three independent experiments with similar results were shown. Right: statistical analysis of cell cycle distribution was shown. *, p < 0.05.

**Figure S5**


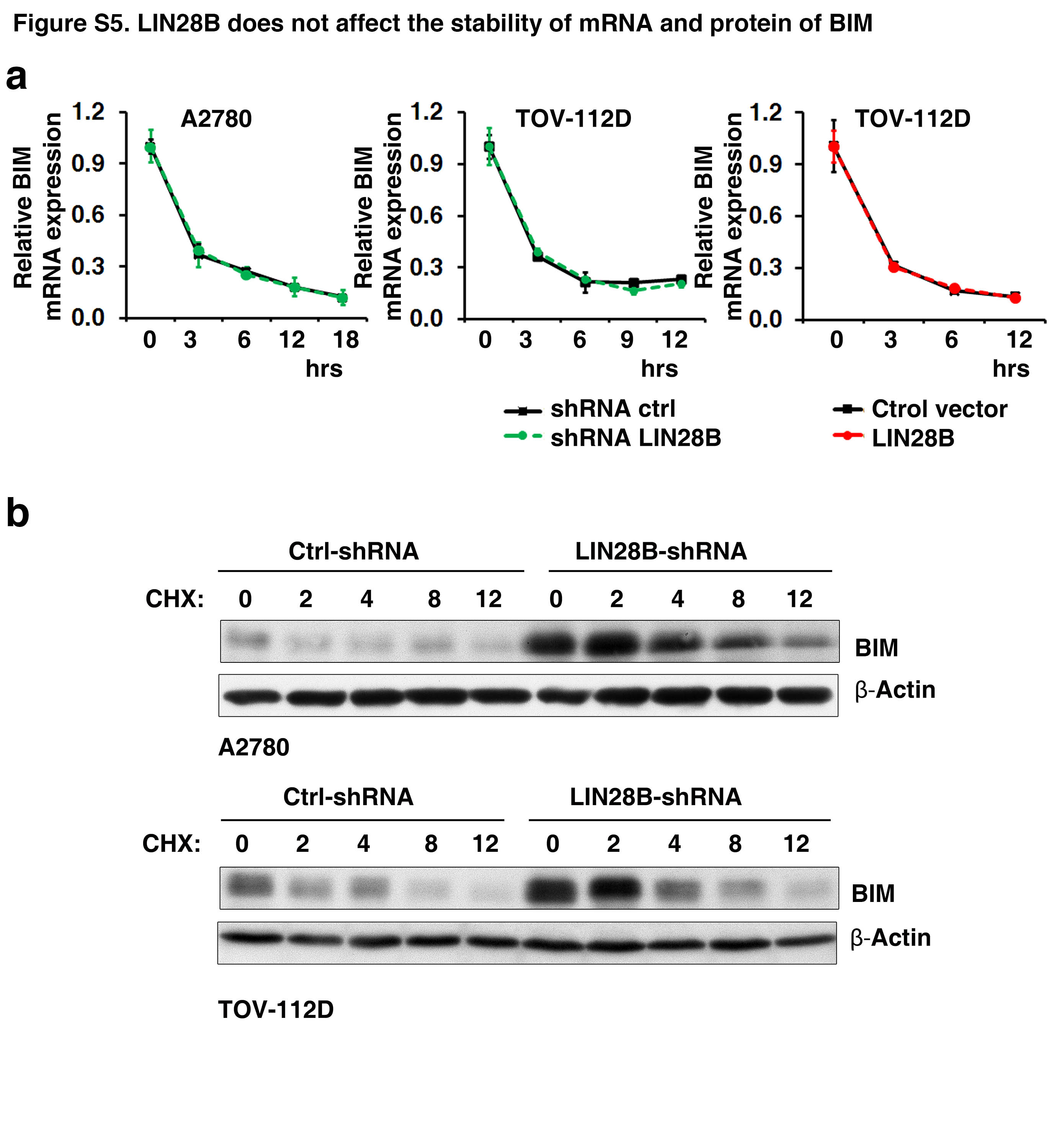


**Figure S5. LIN28B does not affect the stability of mRNA and protein of BIM**

**(a)** mRNA expression level of BIM was detected with qRT-PCR in A2780 and TOV-112D cells (LIN28B knockdown), and in TOV-112D cells (LIN28B overexpression) at 0, 3, 6, 12, 18hrs post-actinomycin D treatment. **(b)** Protein expression level of BIM was detected with Western blotting in A2780 and TOV-112D cells (LIN28B knockdown) at 0, 2, 4, 8, 12hrs post-cycloheximide treatment.
